# A Critical Review on New Approaches for Chronic Disease Prevention in Brazil and Canada: From Wholistic Dietary Guidelines to Physical Activity Security

**DOI:** 10.3389/fcvm.2021.730373

**Published:** 2021-08-30

**Authors:** Juliano Schwartz, Paul Oh, Maira B. Perotto, Ryan E. Rhodes, Wanda Firth, Shannon S. D. Bredin, Alejandro Gaytán-González, Darren E. R. Warburton

**Affiliations:** ^1^Physical Activity Promotion and Chronic Disease Prevention Unit, University of British Columbia, Vancouver, BC, Canada; ^2^Experimental Medicine Program, Faculty of Medicine, University of British Columbia, Vancouver, BC, Canada; ^3^Cardiovascular Prevention and Rehabilitation Program, Toronto Rehabilitation Institute, University Health Network, Toronto, ON, Canada; ^4^West Toronto Diabetes Education Program, LAMP Community Health Centre, Toronto, ON, Canada; ^5^School of Exercise Science, Physical and Health Education, University of Victoria, Victoria, BC, Canada; ^6^Hearts & Health in Motion Program, Nova Scotia Health, QEII Health Sciences Centre, Halifax, NS, Canada; ^7^School of Kinesiology, Faculty of Education, University of British Columbia, Vancouver, BC, Canada; ^8^Institute of Applied Sciences for Physical Activity and Sport, University of Guadalajara, Guadalajara, Mexico

**Keywords:** lifestyle, health, physical inactivity, diet, Brazil, Canada, prevention

## Abstract

In light of new evidence on the prevention of chronic diseases and the elevated rates of overweight and obesity in Brazil and Canada, this critical review aims to interpret and synthesize current aspects regarding dietary and physical activity initiatives in both countries and make future recommendations. The pioneering work presented in the last Brazilian dietary guidelines has been called a model that can be applied globally, given its conceptualization of healthy eating that translates easily to practical guidance. The new Canadian Food Guide has incorporated similar aspects, also putting the country as a leader in dietary guidance. With these new recommendations, citizens in both Brazil and Canada have access to impactful evidence-informed nutritional guidelines. Both documents propose eating patterns that focus not only on health benefits, such as chronic disease prevention, but also incorporate well-being concerning cultural, economic, sociodemographic, biological, and ecological dimensions. A similar approach is required for physical activity to allow individuals to have attainable health and life goals and thereby fully enjoy their lives, regardless of geographical location, health status, and socioeconomic condition, a concept recently described as physical activity security. The wholistic dietary guidelines from both countries represent a change in paradigm in public health. Likewise, national evidence-based policies are warranted to reduce disparities in physical activity, allowing healthier and more active lifestyles for everyone.

## Introduction

As a result of significant improvements in health conditions management, most populations across the world have experienced a decrease in premature mortality related to infectious diseases in the last century (1). At the same time, there has been a rise in different chronic diseases and an increase in consequent morbidity and mortality associated with these medical conditions (2). This shift, called epidemiological transition, is linked to societies' progressive industrialization and extensive automation of manual tasks. Overall, there has been a marked reduction in the requirement for human movement in daily activities, along with widespread availability and active promotion of inexpensive and low-quality food around the world (3). Consequently, in the last decades, high-income as well as low- and middle-income countries (LMIC) have seen their populations engaging in unhealthy lifestyle behaviors, which represent the major cause of chronic diseases and early death (1).

Chronic medical conditions are a leading health problem in high-income countries, such as Canada, which has an estimated population of 37 million people (1, 4). These medical conditions affect LMIC in an even higher proportion—with more than 75% of worldwide deaths from such diseases occurring in these nations (5). This is the case of Brazil, a middle-income country with more than 200 million people, where chronic diseases are also the leading cause of mortality (1, 6).

When the World Health Organization (WHO) launched its global action plan for the prevention and control of chronic diseases 2013–2020, such conditions were responsible for around 63% of worldwide deaths (7). According to the newest report, the last decade has not seen much improvement, and this rate is now about 10% greater (8). Part of this situation is due to the lack of success in tackling these diseases' risk factors, mainly physical inactivity (usually identified when one does not engage in at least 150 min of moderate to vigorous physical activity (PA) per week); unhealthy diet (often characterized when one eats less than five servings of fruits and vegetables per day); smoking (tobacco use in any dose or route); and harmful alcohol intake (commonly observed in irregular or chronic heavy drinking) (3, 4, 6).

Tobacco use has received the most attention among governments and researchers worldwide, resulting in a significant decrease in this behavior in most countries, including Brazil and Canada (1, 9, 10). Global alcohol intake has also had some improvement, leading to stable levels of consumption in the last years—a trend also observed in both the Brazilian and Canadian populations (1, 6, 11, 12). Conversely, there has been an increase in the prevalence of obesity across the globe, which is directly related to unhealthy diet and physical inactivity, thus requiring effective national and international public health measures to tackle these burdens (1, 6, 13). In this respect, new evidence-based documents, such as the current dietary guidelines of both countries and the 2020 global guidelines on PA present cutting-edge advances and along with the newly proposed concept of PA security will be discussed in the following sections.

According to the WHO's latest report, in 2016 there were around 1.9 billion overweight adults worldwide, out of which 650 million were obese (14). While 39% of the adults across the globe have overweight or obesity, in Brazil and Canada this combined overweight/obesity prevalence is 55.4 and 59.8%, respectively (6, 13). As expected, according to official indicators, both countries also face high rates of physical inactivity and unhealthy dietary patterns. In Canada, 83.6% of adults do not engage in at least 150 min of moderate to vigorous PA per week, whereas in Brazil this prevalence is of 44.8% of the adult population (4, 6). This substantial difference is likely due to the lack of agreement between the methods of assessment used in each country. In Canada, PA was measured with accelerometers, which present better reliability and sensitivity, whereas Brazil used self-reports, which usually overestimate PA (15). To illustrate this difference, Colley et al. (16) compared both methods, which were used in a Canadian survey in 2016, showing that almost half of the Canadian adults reported engaging in 150 or more minutes of moderate to vigorous PA per week, whereas the accelerometer-measured data showed that this was the case for only 17% of these individuals. Regarding diet, 71.3% of Canadian adults and 87.1% of adults in Brazil do not eat five or more fruits and vegetables daily (4, 6).

Considering these realities, and in light of the new concept of PA security as well as innovative approaches introduced in the latest Canadian and Brazilian dietary guidelines and in the latest WHO guidelines on PA, this critical review aims to present and discuss current aspects regarding chronic disease prevention in adults in Brazil and Canada. Specifically, we analyze and synthesize current preventive approaches in terms of PA and diet carried out in each country, with a particular focus on PA, and make future recommendations.

## Prevention Approaches in Brazil and Canada

Given the alarming prevalence of unhealthy diets and insufficient levels of PA in Brazil and Canada, both countries have implemented population health programs and protocols to address these risk factors, including some novel initiatives (17–20). An innovative action taken by both countries was the update of their dietary guidelines. Brazil led the way, with their revolutionary approach to inform healthy eating (21). Canada followed with the launch of their new Food Guide, which incorporated similar concepts (22). Both documents address the context of eating rather than the usual focus on nutrients and food groups, which was a turning point in the field of nutrition and public health (23, 24). A common specific area of concern relates to the science regarding the negative impact on health from processed and ultra-processed food (22). With such evidence, the latest version of each guide addresses the importance of avoiding highly processed food and prioritizing minimally processed and unprocessed options (25, 26). These recommendations are directly aligned with the focus on chronic disease prevention, a primary goal in both documents (17, 20).

Without the food industry's participation in their development, both guidelines provide clear alternatives to processed and ultra-processed food, using simple and direct messaging (25, 26). Good examples are the recommendations to eat plenty of fruits and vegetables and to have water as the beverage of choice. Instead of the widely used nutrition-based guides, these food-based guidelines focus on healthy eating and, at the same time, make a direct ecological impact by encouraging a sustainable eating pattern (17, 21).

Another aspect present in both guides is the emphasis on meal planning and the connection with others during the act of eating, which contemplates sociocultural values, and are also considered strategies to support a healthy diet (23). With its emphasis on sustainability, this food- and meal-based wholistic approach put Brazil and Canada in the global leadership of dietary recommendations for optimal health of both human beings and the planet (27).

As is the case with all population-based guidelines, there is incremental work to be done beyond their release (28). There is a need for accompanying implementation tools for the guide, policy development, and multiple layered initiatives and support to target health behavior change (26). There are many barriers to be overcome, and it can be challenging to make healthy food choices (21). The Brazilian and Canadian dietary guidelines recognize the influence of social and physical environments, household income and the many determinants of health, educational and health programs, along with government food policies for the successful implementation of these guidelines (25, 26, 29).

While Brazil's latest dietary guidelines have been widely praised (30), by the time of its publication the country took a surprisingly different approach in respect of PA guidelines, despite the opinion of Brazilian researchers involved with the promotion of PA and health. The efforts and costs involved in creating national guidelines as a policy for health promotion were considered unjustified, and the country instead chose to adopt the general WHO PA recommendations (31).

Conversely, investments in PA policies are a priority in Canada (32, 33), which has led the country to be a leader in the promotion of the health benefits of PA and the development of evidence-informed PA recommendations for healthy individuals and persons living with chronic medical conditions (34–39). This includes leading international initiatives and conferences, such as the first and second “International Conference on PA, Fitness and Health” (held in 1988 and 1992), the “Dose-Response Symposium” (October 2000), the “Communicating PA and Health Messages Science into Practice” meeting (2001), the Toronto Charter for PA (2010), and the International Society for PA and Health Congress (2010 and 2021) (36, 40, 41). Also, the Canadian government was one of the main supporters of the 2020 WHO PA guidelines, which was launched in light of substantial advances in the field after the release of the previous version (42).

Besides an emphasis on participation and inclusivity, aiming at a broader reach, the latest WHO guidelines on PA address the new science on activities' intensity and duration (43). Specifically, the document highlights the importance of light-intensity PA and states that some activity is better than none. This echoes previous statements made by Canadian and international experts highlighting the importance of simply becoming more active (44–46). Also, due to insufficient supporting findings, these guidelines have removed the requirement for PA to be performed in bouts of at least 10 min of moderate to vigorous intensity to obtain health benefits (43). This new evidence is critical for less affluent settings since regular recreational PAs are inaccessible for many in these locations, as is the case in some areas in Canada as well as LMIC in general, including Brazil (47–49). Indeed, discrepancies in PA participation have been widely observed, with socioeconomically advantaged individuals having more opportunities to be physically active (50, 51). The inclusive approach presented in the 2020 WHO PA guidelines therefore has the potential to impact health outcomes in both Canada and Brazil, since inequalities in PA, besides contributing to the pandemic of physical inactivity, were found, for example, to be predictive of obesity in middle- as well as in high-income countries (51, 52). Further work is warranted, however, owing to the limited uptake of PA guidelines internationally and the deficits-based nature of PA messaging that focuses on the health perils of too little PA (i.e., increased risk for cardiometabolic disease) rather than the diverse benefits of simply moving more (53, 54).

Numerous disparities across the world require PA to be approached in terms of equity. This view is presented in the new Brazilian and Canadian dietary guidelines (25, 26). Just as these documents should lead to the development of strategies to address inequity and food insecurity, PA policies should also lead to recommendations on equity. This concept was recently described as PA security—when everyone, everywhere, would have unrestricted access to PA, meeting all needs for an active and healthy life (55). Aligned with this are two research priorities established in the latest WHO guidelines on PA (43). One of these concerns is the need for more evidence from LMIC and economically disadvantaged communities since most of the science on PA is based on findings from high-income settings. Another research topic to be prioritized refers to the health benefits of the aforementioned light-intensity PA. Both aspects relate to realities in Brazil and Canada since much of the PA carried out in different locations of these countries is of light intensity (48, 56). In fact, there is a pressing demand for PA security in certain areas of Canada and in much of Brazil's territory, where utilitarian PA, such as active commuting, is one of the main forms of PA (57, 58). Importantly, although some of these regions present a high prevalence of PA, many times such engagement happens due to a lack of alternatives, rather than simply choice, including walking and cycling to and from work/school in unsafe traffic areas as well as in locations with a high perception of criminality (55, 59, 60).

An important point corroborating this new approach is the evidence demonstrating significant health benefits with lower levels of PA than suggested in different guidelines (49). It has been demonstrated that such benefits can be attained even with half of the usual recommendation, particularly among previously inactive individuals (46). These findings hence show the lack of a minimum threshold in the amount of PA for health gains—a position which has just been endorsed by the WHO (42). This has a great impact in terms of public health, since engaging in regular PA, regardless of the intensity and the duration, can promote change in other health behaviors, such as diet and smoking, thereby significantly contributing to the prevention of chronic diseases (61, 62).

Moreover, despite the well-known health benefits of regular PA (46), additional positive effects on the immune system, mental health, and quality of life have also been reported in the context of the COVID-19 pandemic, thus providing further support for PA to be made accessible to everyone (63). Therefore, the current moment is a window of opportunity to emphasize PA as a human right, calling for actions from local policy- and decision-makers (55, 64). In light of this recent evidence, the following section addresses aspects of different dimensions that should be considered in contemporary initiatives in Brazil and Canada aimed at population PA promotion. In addition, the Discussion section addresses more in-depth different initiatives carried out in each country.

## Macro-Environmental Dimensions of Physical Activity

PA dimensions are commonly addressed only in terms of individual and physiological characteristics, such as energy expenditure, as well as activities' duration, frequency, and intensity, characterizing a strictly biomedical relationship (65, 66). At the same time, internal aspects such as motivation and self-regulation are frequently identified as significant barriers to PA in high-income nations (47, 67). However, given the low rates of physically active individuals in countries like Canada (68), it is unlikely that lack of motivation alone explains the level of physical inactivity in industrialized countries, and consequently other characteristics of population subgroups should also be considered. In fact, PA interventions focusing only on personal factors were excluding for many, particularly in disadvantaged areas, regardless of the geographic location (47, 59). These findings suggest the need for a more socially contextualized approach to make PA an attainable and enjoyable purpose for everyone, including those in middle- and high-income countries (51, 69). In that regard, some of the aspects employed in the current Brazilian and Canadian dietary guidelines could be used as references, aiming at establishing a wholistic approach to PA guidance that would influence research and policy development for equitable interventions for PA promotion (70, 71). The name of each of the following dimensions under which PA should be seen is not necessarily the only option since some have interchangeable characteristics for adults. The emphasis here is on the overall content presented rather than the specific label of each dimension.

### Cultural

The interest in understanding the influence of culture on PA behavior is not new, however, to date, still little is known about this topic (72, 73). Commonly, health promotion interventions, including those aiming to increase PA participation, are based on outcomes from studies conducted with certain ethnicities and are then generalized to all cultures. As a consequence, since not all these interventions are universally transferable, individuals with different traditions, such as those in specific ethnic groups, religious communities, immigrant clusters, and indigenous peoples often present lower levels of PA (74–76). Even when resources are available and the built environment is conducive to PA, the absence of cultural care can limit changes in this behavior (77, 78). Accordingly, an increasing number of studies report that initiatives aiming at raising PA levels should take cultural backgrounds into consideration (64, 76, 79).

Despite Brazil's culture being a result of the integration of distinct nationalities and ethnic backgrounds, which implies a population full of diverse cultural roots (80, 81), only a few studies have investigated Brazilian initiatives that incorporated cultural contexts. These include an extensive intervention considering differences between cities in one specific state (82), and one community-based program addressing traditional components of specific PAs (83). Given the large and very heterogeneous population of the country, there is a need for a broader emphasis on cultural aspects in PA interventions. This is supported by studies showing a high prevalence of overweight and obesity in indigenous peoples, a higher risk of obesity among immigrants with higher levels of acculturation, and a high prevalence of physical inactivity in black individuals (81, 84, 85).

Conversely, in Canada, where only one third of the population reported being of Canadian origin (86), more studies have investigated PA interventions considering cultural backgrounds than in Brazil (87–90). However, there has been a growing movement calling for a more systematic approach in the country to implement culturally safe PA programs, similar to measures already in place in other health promotion initiatives (91). Although such approach focuses initially on indigenous peoples, it could be extended to other ethnicities, since the indigenous population corresponds to around 5% of Canadians (86), and some individuals from different ethnicities report not feeling culturally supported in PA initiatives (74, 92). For instance, Culp (93) highlighted the importance of addressing immigrants' and ethnic minorities' cultural norms in order to reduce PA barriers. Also, Brooks-Cleator and Giles (94) showed that even PA programs aiming at being culturally relevant for indigenous people have some aspects not meeting this purpose. This demonstrates the overall importance of research and promotion of culturally sensitive PA interventions contemplating all ethnicities (76).

### Economic

Following PA guidelines requires different investments that are not affordable for various individuals (47, 95). Despite the direct expenditure of money required to participate in some activities, related for example to the purchase of clothes, allocating a certain number of minutes per day or week exclusively to engage in PA is an investment not compatible with the routine of many (63, 96–98). It is not surprising that modifying one's routine to include PA was shown to be impractical due to different reasons, such as tiresome jobs, long commutes, holding more than one job and/or also studying, and competing housework chores and/or family responsibilities (47, 99). Although different barriers to PA are found in specific regions (95, 100), low income is associated with PA insecurity in high- as well as lower-income countries (47, 101).

Whereas, in some high-income countries the main obstacle for leisure-time PA is lack of time and possibly motivation—mainly for individuals of high socioeconomic status—in Brazil the main barrier is lack of money (98, 99, 102). Despite presenting some advances in economic welfare, Brazil is still one of the world's most unequal countries (59). This is reflected, for example, in higher levels of physical inactivity in low-income groups than in the economic elite, which has more means to afford being physically active (99). To address this and other inequalities related to chronic disease prevention, important actions were taken, mainly between 2004 and 2013, such as the inclusion of PA in the national health promotion agenda (29). However, although more than 40 million people were lifted out of poverty during this period (103), the challenge to reduce economic inequality in PA is substantial. In 2013 about 50 million Brazilians were still poor or extremely poor (104), and in 2019 around 60 million individuals were living in poverty (105).

Currently, economic aspects can be considered the main obstacle to be overcome in order to reduce disparities in Brazil, as evidenced by the significant hardship faced by some sectors, such as health and science (106). This is a result of changes that have been implemented in the national economy since 2016, with direct effects on physical inactivity and its consequences (59, 107). In 2017, for example, after years of stability, there was an increase in hospitalizations due to affective disorders, coinciding with stagnant PA levels in the same year, after increasing between 2006 and 2016 (108). Such economic changes deepened national discrepancies in PA, with less economically advantaged regions presenting a higher burden of mortality related to chronic diseases due to low PA levels (107). Other findings demonstrate the complexity around the difficulties faced by financially disadvantaged Brazilians. For instance, according to Galvim et al. (96), even when free PA programs are offered to economically vulnerable communities, adherence is low. They also report a significant number of dropouts, mainly due to participants finding new employment. Additionally, in contrast to high-income areas, solely building free of cost fitness facilities in low-income neighborhoods appears to be insufficient to favor PA in these communities (109). Therefore, more innovative initiatives are required, allowing for broader and more effective participation (2, 59). In that regard, several investigations have shown how increases in PA levels can lead to considerable savings in the country, thus justifying that investments in policies for the promotion of an active lifestyle must be a priority, even in this adverse context (5, 107, 110, 111).

As a high-income country, economic factors may be regarded as less limiting for PA engagement in Canada. However, longitudinal data starting in the twentieth century has shown that, through the decades, individuals with low income were constantly engaging less in leisure-time PA, and therefore this group should be prioritized in terms of efforts to increase population PA in the country (112). This is corroborated by findings showing that natural and free options for engagement in PA during the winter in Canada, such as an urban trail on a frozen waterway, which could help minimize weight gain during this season, are usually not used by low-income adults, likely due to access limitations using public transit as well as lack of adequate clothing (113). Additionally, Luan et al. (114) showed that even utilitarian PA, such as active transportation and its associated benefits—including decreases in body weight—is less accessible for low-income Canadians, highlighting the fact that some interventions to increase active travel may actually increase inequalities since many initiatives favor financially advantaged individuals in a higher proportion.

Overall, economic disparities in PA are present in different parts of Canada, such as recently reported by physicians and policy experts who serve low-income communities (47). These stakeholders stated that, given the difficulties in meeting national PA guidelines, such recommendations were not relevant for the 3.5 million Canadians living in poverty. Thereby, since there is a clear need for more actions to make PA accessible to members from low-income households in the country, such initiatives could take advantage of the savings that a more active population cause in health care (115). This is corroborated by the fact that in Canada, physical inactivity was found to be the primary unhealthy behavior leading to costs in the health sector (116).

### Sociodemographic

Several studies have reported gender inequalities in PA programs, with a higher overall prevalence of inactivity in women than in men, thus signaling the need for gender-neutral interventions and actions that properly contemplate each gender (50, 79, 117, 118). For instance, according to Althoff et al. (52), obesity is more strongly predicted by gender disparities in PA than by physical inactivity alone. Similarly, using data from 142 countries, Mielke et al. (118) showed that small decreases in physical inactivity in women would lead to an overall 10% reduction in this unhealthy behavior, achieving a global target set by the WHO (79), even without changing the prevalence in men. Besides these disparities between men and women, education and marital status were other sociodemographic aspects investigated in different studies, presenting interesting associations with PA. Although most studies found that more highly educated individuals have higher levels of PA (6, 119–122), some investigations found opposite results (117, 123). The association between marital status and PA seems to be more complex, with some studies showing that married individuals are more active (50, 100, 124), some research showing the opposite (121, 122, 125), and some studies indicating no significant difference (117, 119, 126).

Some gender differences are present in PA engagement of the general Canadian population (68), as well as of specific groups, such as indigenous peoples (127) and immigrants (75), with studies consistently reporting that men were more physically active than women. In regards to education, similarly to most international findings, Canadians tend to be more physically active with increasing education levels (119, 122). With regards to marital status, two studies found that married people are more physically active (128, 129) while two studies showed the opposite (119, 130).

In Brazil, sociodemographic inequalities in PA are considerable, particularly with regards to recreational activities. In terms of gender, men engage more in PA than women. This trend is observed not only in the general population (6) but also in rural communities (131) and among black individuals (81). Brazilians are also more physically active the more years of schooling they have (6), but the findings are mixed with regards to marital status. Whereas, one study showed that married individuals were more active (100), two studies showed the opposite (132, 133), and three others did not find any difference between married and not married individuals (131, 134, 135).

### Biological

Many of the first population-based initiatives on PA targeting adults did not contemplate certain groups, such as the elderly, pregnant women, and individuals living with chronic diseases (136, 137). Overall, the first interventions focused on adulthood used to address only young and middle-aged adults (138, 139). However, with the inversion in the age pyramid, the older population has been receiving increasing attention, with several guidelines and other PA initiatives being tailored to older adults (140, 141). The WHO guidelines on PA have a specific chapter for this population (43). Besides recommending a minimum of 150 min of moderate to vigorous PA, the document mentions the importance of light-intensity activities and states that some PA is still of benefit for health in case the recommendations are not fully met by this group.

Additionally, for the first time, the 2020 WHO guidelines have addressed pregnancy and morbidities (42). The document provides specific recommendations for pregnant and postpartum women, and has dedicated sections for both chronic medical conditions and disabilities (43). The first section addresses cancer, hypertension, type 2 diabetes, and HIV, which is also considered a chronic condition given its treatment's advances and availability. And the other section focuses on multiple sclerosis, spinal cord injury, and cognitive function impairments, including Parkinson's disease, stroke, depression, schizophrenia, and intellectual disabilities.

Individuals in these groups need comprehensive and permanent initiatives, to allow a safe and continuous engagement in PA. In this regard, good examples of simple and effective strategies to reduce barriers for these individuals are the Canadian evidence-based pre-participation screening tools Physical Activity Readiness Questionnaire for Everyone (PAR-Q+) and the electronic Physical Activity Readiness Medical Examination (ePARmed-X+), which are currently being translated and adapted to different languages (142, 143). The Brazilian version of the PAR-Q+ has just been validated (144).

### Ecological

The effects of different human actions have posed severe risks to the planet, and the consequences can be disastrous (145). Therefore, measures to mitigate these effects and avoid further harm are deeply needed. In that regard, recent evidence has revealed the positive impact that interventions to increase PA levels can have on the natural environment (62). This is aligned with a global action plan on PA, highlighting the contributions of an increase in active recreation, sports, walking, and cycling to a sustainable and prosperous world (145). Reductions in traffic volumes and speeds, along with improving infrastructure to provide safe and welcoming spaces for active transportation, lead to a decrease in traffic accidents and pollution (146). Other incentives to active commuting, such as bike-sharing programs, reduce automobile use and decrease fossil fuel consumption, thereby contributing to the mitigation of climate change (69).

Another influence of PA on planetary health is related to dietary patterns. Given the deleterious effects of packaged highly-processed food on the environment, a reduction in its consumption is greatly warranted (24). In this respect, replacing these options with unprocessed foods, such as fruits and vegetables, contributes to a healthier weight and preserves planetary resources (147). Besides the fact that eating these plant-based alternatives in combination with PA leads to better weight management, physically active individuals usually eat more fruits and vegetables (62).

Additionally, aside from investigations addressing the built environment in the last decades, recent studies have analyzed the relationship between PA and the natural environment in Brazil and Canada. This topic is critical in both countries since they are among the top 10 nations in greenhouse gas emissions (148). A positive aspect in that regard is that Canadians cited their concern with the planet as one of the reasons to engage in PA (149). However, Galway et al. (149) also identified some issues that need to be addressed in the country, such as the underutilization of cycling as a mode of transportation and the barriers related to winter weather. In their review on cycling usage, including studies from Canada and Brazil, Jahanshahi et al. (150) highlight some issues, such as the need for initiatives addressing inequalities in accessing bike-sharing systems. On the other hand, according to Benedini et al. (151), bicycle infrastructure expansions has led underrepresented groups in Brazil to cycle more. As mentioned before, this is likely related to the fact that active transportation is the only commuting option for several individuals in low-income settings, which can be linked to the precarious transit system and spatial segregation in several regions of the country (58).

## Discussion

Mirroring the remarkable progress regarding national dietary guidelines and related policy development in Brazil and Canada, emergent evidence indicates that similar approaches are necessary in terms of PA (2, 55, 57). Findings from the last decades have also highlighted the importance of strategies to target sedentary behavior, which is different from physical inactivity (152). Whereas, physical inactivity is usually defined as not meeting international PA recommendations, sedentary behavior relates to the time spent in activities that require very low levels of energy expenditure in prolonged sitting, reclining or lying postures, such as watching TV, using the computer, and playing video games (43, 46, 153). Accordingly, the updated global guidelines have acknowledged the relevance of this theme, and the new document addresses both PA and sedentary behavior in several sections as well as in the title of the document: WHO Guidelines on PA and Sedentary Behavior (43). The WHO also recognizes that broader and more inclusive initiatives are needed to increase proper access to PA and reduce sedentary time, addressing internal and external barriers, regardless of cultural background, education level, gender, age, socioeconomic conditions, and health status, while promoting sustainable development (145, 154).

Since the health risks associated with too much sedentary time add to the risks related to insufficient PA, specific strategies are required to decrease the engagement in activities such as passive screen-time (155). Strategies applied to break long sitting periods include the use of electric adjustable-height desks that alternate between sitting and standing positions (156), as well as treadmill desks (157) and bike desks (158). However, the aforementioned options are not accessible for many, are not environmentally sustainable, and are not practical and/or appealing enough for several people (62, 159, 160). This situation requires alternative approaches, such as for example, walking meetings, which has been reported to also lead to social benefits (161), as well as some recently proposed solutions with promising results, including intermittent upper body ergometry, an attractive option for those who prefer to remain in the seated position (162), and low-cost standing desks, which are made out of cardboard, are suitable for use in different locations such as at home, and can have an inexpensive and environment-friendly recycling process (163, 164). Despite the challenges involved in implementing public policies that contemplate widespread access to PA promotion and sedentary behavior reduction initiatives, especially in low-income settings, localized comprehensive interventions have been recently implemented in many countries, including Canada and Brazil, and must be scaled up (60, 101, 146, 165).

Although disparities in PA are still present in Canada, different efforts have been made to tackle this situation, including the development and implementation of several provincial and national PA policies (33, 166). An example was the inclusion of accelerometer-measured PA in the Canadian Health Measure Survey (68). PA monitoring systems allow the identification of target groups, the assessment of the population impact of policies, and the detection of changes in PA related to policies, thereby guiding effective actions to increase PA levels (167). Country-wide initiatives to reduce sedentary behavior and increase physical activity levels have addressed physical literacy—knowledge and skills necessary to take responsibility for a long-term engagement in PAs—as well as health literacy—the capacity of an individual to make proper health decisions (168, 169). This is the case of the ParticipAction program, a non-profit organization aimed at making daily physical activity a vital part of Canadians' lives (166), and the federal initiative Let's Get Moving—A Common Vision for Increasing Physical Activity and Reducing Sedentary Living in Canada (33). Both initiatives promote education material and activities to increase health literacy in relation to physical activity, as well as campaigns and products for community mobilization and engagement, such as the case of a recent free mobile phone app (19, 170). Specifically, the Let's Get Moving initiative encourages municipalities, volunteer associations, faith-based groups, and service clubs to take into account the experiences of individuals and their perspectives on what being active means, an approach directly related to health citizenship (33, 171). Another example is the Physical Activity Line (PAL), developed by researchers at the University of British Columbia to provide telehealth support (172). Residents from anywhere in the province were able to receive free evidence-based PA guidance from a qualified exercise professional via the telephone or internet. The PAL led to a major advancement in the promotion of health benefits of PA and served as a role model in the area of telehealth across Canada. The PAL was incorporated into HealthLink BC where it is now a part of the Government of British Columbia's menu of telehealth services (173). Other initiatives include the National Health & Fitness Day, and the Canadian Health & Fitness Institute, aimed at promoting active citizenship (174), as well as the National Indigenous PA Awareness Week, organized by the Indigenous PA and Cultural Circle, with a specific focus on this population (175).

Unlike in Canada, inequalities in Brazil are much more widespread and have lately been increasing (59). Nevertheless, with several cutting-edge researchers on PA promotion, some initiatives in the country have received worldwide recognition (176, 177). Examples include the World PA Day, adopted in the five continents, and the Agita São Paulo program (18). The latter is a permanent PA intervention focused on chronic disease prevention, known for its campaigns targeting sedentary behavior and physical inactivity, and for the participation of numerous and diverse communities, which led the WHO to considered it a model for other LMIC (176). More recent programs in primary health care for PA promotion and sedentary behavior reduction have interesting proposals. The Health Gym Program and the Expanded Family Health Center are national health promotion strategies that make use of public spaces to offer physical activity in different communities (178, 179). These actions are commissioned to health professionals along with the community in each location, and include education activities such as lectures, and different groups of PA sessions, such as walking and other aerobic activities as well as resistance training (179, 180). The Sport and Leisure in the City is a more inclusive program, which includes individuals with disabilities (181). Another example is the Family Health Strategy, which provides multidisciplinary teams to promote counseling for physical activity among other health aspects (182). Although the implementation of these different initiatives has faced substantial challenges, significant positive outcomes have been reported, including savings in the public health system and increases in physical activity levels (183–185).

The first version of the present study recommended the establishment of PA recommendations specifically tailored for the Brazilian population, highlighting that, based on the aforementioned experiences, Brazilian researchers had the required expertise for the development and implementation of effective national guidelines on PA. Exactly five days after the current study has been submitted for publication we learned that the PA Guidelines for the Brazilian Population had just been launched (186). The review process of our manuscript allowed us to acknowledge this publication, which brings significant messages, such as the emphasis on keeping in mind that engaging in any PA, whenever and wherever possible is better than nothing and can lead to health benefits. Additionally, the document makes clear that engaging in PA does not depend solely on one's decision, and that several factors may act as barriers or facilitators, such as personal, environmental, cultural, financial, and political factors. The guidelines recommend Brazilians to have conversations about these topics, and to approach municipal, state, and federal politicians from different sectors, to enquire about how their communities can be more propitious to PA engagement (186). Thereby, these guidelines explore health citizenship, by addressing entitlements and responsibilities in the political and social developments of public health practices, involving each citizen as well as the government for the successful uptake of the guidelines (171).

This approach is in agreement with the WHO document Global Action Plan on Physical Activity 2018–2030: More Active People for a Healthier World, which states that not recognizing and investing in PA as a priority is a serious mistake, leading to harmful consequences on the health system, economic development, and the environment, as well as quality of life and community well-being (145). Indeed, despite the importance of the aforementioned initiatives, considering Brazil's large population and the current context of significant inequalities, developing national PA guidelines and providing the support of appropriate public policies for its implementation is crucial to prevent setbacks and to enable massive participation (18, 59). Therefore, with the WHO making the recommendation to compare the health outcomes of PA interventions in high and LMIC, it is the perfect timing for Brazil to launch its national guidelines (187).

Additionally, since the new evidence shows that policies to prevent chronic diseases should not be limited to improving individual's health behaviors but also focusing on reducing several inequalities, there is a need for the training of different professionals to put such policies into action (55). According to Lambert et al. (55), this includes transport engineers and urban planners, as well as PA providers in the health sector, such as qualified exercise professionals, who should be well prepared to deliver socially contextualized PA interventions. However, health agents in charge of promoting PA in the Brazilian Unified Health System reported a lack of knowledge and protocols to properly do so (188), which emphasizes the importance of the newly released PA guidelines in the country.

## Conclusion

Canada followed the pioneer work of Brazil, in launching innovative dietary guidelines. Besides targeting chronic disease prevention, both documents also apply a wholistic approach, addressing economic, biological, sociodemographic, ecological, and cultural dimensions. As well, Brazil and Canada are developing and implementing policies to help support the uptake of their respective dietary guides. Similarly, broader and more inclusive initiatives are needed to allow proper PA access in both countries. Although some PA disparities still persist in Canada, the newest evidence shows that Brazil's population would greatly benefit if the country could follow the Canadian lead in prioritizing investments in PA policies. The launch of the brand new PA Guidelines for the Brazilian Population is a significant step in this respect. In addition to individuals in both countries having a clear guidance to help them to decide what to eat and how, they should also have appropriate guidance to support their entitlement to be physically active and thereby enjoy the numerous advantages linked to this human right.

## Author Contributions

JS, PO, RER, and DERW conceived and designed the study. JS searched and read the literature, and drafted the manuscript. PO, MBP, RER, WF, SSDB, AG-G, and DERW critically revised and provided insightful edits to the manuscript. All authors reviewed and approved the submitted version.

## Conflict of Interest

The authors declare that the research was conducted in the absence of any commercial or financial relationships that could be construed as a potential conflict of interest.

## Publisher's Note

All claims expressed in this article are solely those of the authors and do not necessarily represent those of their affiliated organizations, or those of the publisher, the editors and the reviewers. Any product that may be evaluated in this article, or claim that may be made by its manufacturer, is not guaranteed or endorsed by the publisher.
